# Commentary in response to BMC Urology publication entitled “Infection-related hospitalization following ureteroscopic stone treatment: results from a surgical collaborative”

**DOI:** 10.1186/s12894-021-00915-3

**Published:** 2021-11-06

**Authors:** Wesley A. Mayer

**Affiliations:** Baylor College of Medicine, Houston, TX USA

## Abstract

This Commentary is in response to the BMC Urology publication entitled “Infection-related hospitalization following ureteroscopic stone treatment: Results from a surgical collaborative”. This study utilized a registry with prospectively recorded standardized data elements named Reducing Operative Complications from Kidney Stones, part of the Michigan Urological Surgery Improvement Collaborative, to identify risk factors of infection-related hospitalization after ureteroscopy for stone treatment. The study included 1817 primary URS procedures for urinary stones in 11 practices in Michigan. They found 43 patients (2.4%) were hospitalized with an infection-related complication and 3 patients died during their hospitalization (0.2% mortality rate). Just over 20% of patients did not have a pre-operative urinalysis or urine culture, representing a deviation from guideline recommendations. Also, in the hospitalized group, none of the 12 patients (27.9%) who had a positive pre-operative urinalysis or urine culture received pre-operative treatment. A multivariable analysis identified higher Charleston Comorbidity Index, history of recurrent urinary tract infection, increasing stone size, intraoperative complications, and fragments left in-situ as independent risk factors for hospitalization from an infection after ureteroscopy. This commentary discusses caveats to the data as well as short-comings of the study. It also reviews more broadly infection after ureteroscopy, includes findings from similar studies, and highlights guideline recommendations to reduce infection risk.

Commentary:

A systematic review from the European Association of Urology (EAU) Section of Urolithiasis including over 24,000 ureteroscopy (URS) procedures from 14 large studies found an overall infectious complication rate of 3.9% [1]. Urosepsis was found in 0.51% of the cohort. The economic burden of sepsis totals over 24 billion dollars a year in the US alone [2]. Moreover, the mortality rate from URS ranges from 0.04% from the Clinical Research Office of the Endourological Society (CROES) Ureteroscopy Study Group to 0.15% in the EAU systematic review [1, 3]. Clearly, mitigating the risk of infectious complications after URS is an essential quality improvement goal in Endourology.

The Michigan Urological Surgery Improvement Collaborative (MUSIC) is a physician-led quality improvement (QI) collaborative that includes academic, private practice, and community urologists across the state of Michigan [4]. Their work has resulted in 40 publications and has served as a model for quality improvement consortiums in urology in the United States. Although originally focused on prostate-related QI, their focus expanded to include kidney stone surgery and more recently renal masses. Now more than 150 urologists across Michigan are contributing data for Reducing Operative Complications from Kidney Stones (ROCKS).

In the current study, the authors used the ROCKS registry to identify 1817 URS procedures in 1737 patients undergoing primary URS for urinary stones in 11 practices in Michigan [5]. Abstractors prospectively record standardized data elements including patient and stone characteristics, surgical details and complications. Through chart review, the authors identified patients hospitalized within 30 days with an infection-related complication. They found 43 patients (2.4%) were hospitalized with an infection-related complication and 3 patients died during their hospitalization (0.2% mortality rate). Interestingly, just over 20% of patients did not have a pre-operative urinalysis or urine culture (PUC) and there was no difference in this number between the hospitalized group and the non-hospitalized group. In the hospitalized group, 12 patients (27.9%) had a positive PUC (defined by either positive nitrites on a urinalysis or a positive culture) but none of them received pre-operative treatment. In comparison, there were 266 patients (15.4%) with a positive PUC in the non-hospitalized group and still only 59 of them received pre-operative treatment (22.2%). These differences approached but did not reach statistical significance.

A multivariable analysis identified higher Charleston Comorbidity Index, history of recurrent urinary tract infection (UTI), increasing stone size, intraoperative complications, and lithotripsy with fragments left in-situ as independent risk factors for hospitalization from an infection-related complication after URS. The authors appropriately recognize the limitations of an extensive multivariable analysis with a small number of events. Pre-operative alpha blockade was also identified as a factor decreasing the risk of hospitalization from infection-related complications; however, this did not hold true on multivariable analysis. The reason for the alpha-blocker therapy was not recorded so this could represent a lower risk group who were more likely to be followed on medical expulsive therapy. This is just one example of the types of confounding variables present in analyses such as these.

There are several important caveats to the data presented in this study. Patients were over 18 years of age undergoing unilateral URS for urinary stones. Anyone with an ipsilateral nephrostomy at the time of URS, or who underwent URS after percutaneous renal surgery were excluded. Thus, the results are not generalizable to these groups of patients, pediatric patients, or patients undergoing bilateral URS. Also, hospitalization for infection-related complications was identified through retrospective chart review based on systemic inflammatory response (SIRS) criteria. The causes for admission due to infection after URS can be variable and the authors appropriately recognize that more patients may have been hospitalized without SIRS criteria that were not captured. Furthermore, this analysis does not endeavor to address the broader issue of infection risk after URS including those instances that do not result in hospitalization but cause an increase in phone encounters and office visits.

The authors also appropriately recognize the inherent limitations of the analysis based on the registry data collection tool. For example, the collection tool did not capture data on the type of laser lithotripsy technique utilized. Thus patients undergoing a “dusting” technique would have likely been coded as having fragments left in-situ, regardless of the size of fragments. This could make “fragments left in-situ” a surrogate for stone size. Also, information on stone cultures was not collected. Stone cultures have been shown to be better predictors of sepsis and SIRS than voided cultures [6, 7]. Eswara et al. reported that urine cultures were only positive in 7% of patients, whereas stone cultures were positive in 29% in patients undergoing URS [8]. In that study, the overall sepsis rate was about 3–4% for all patients, 8% for patients with positive stone culture, and only 1% for those who had a negative stone culture. Finally, the data collection tool did not capture use of ureteral access sheaths (UASs). Traxer et al. examined prospectively collected data from more than 2000 patients around the world treated with URS and found a decrease in the rate of infectious complications with the use of UASs, although the reason for UAS usage was not recorded [9].

Limitations aside, this study revealed that over 20% of their patients undergoing URS did not have either a pre-operative urinalysis or urine culture (PUC). This is a surprisingly prevalent deviation from the standard of care as recommended in both the AUA Guidelines on Surgical Management of Stones and the EAU Guidelines on Interventional Treatment for Urolithiasis [10, 11]. Carlos et al. also found that adherence to guidelines concerning antibiotic administration before PCNL and URS with a negative PUC varies by scenario and provider [12]. In response to an Endourology Society survey, 21% to 28% reported antibiotic use before a URS that is not consistent with recommendations from the AUA and EAU for patients with a negative PUC. In terms of a positive PUC, nearly all surveyed provide pre-operative antibiotics. However, data from this current study shows this may not hold true in practice. With that being said, prospectively collected data from 462 consecutive patients in a single National Health Service institution in the United Kingdom demonstrated that a positive PUC was significantly associated with post-operative urosepsis on multivariable analysis despite appropriate treatment with a pre-operative course of antibiotics (OR 4.88) [13].

The current study reinforces many previously described risk factors and predictors for hospitalization from infectious complications after URS in a diverse cross-section of patients and practice environments. In the aforementioned EAU systematic review, the most commonly identified risk factors were higher comorbidity scores, recurrent UTIs, larger stones, and positive PUCs [1], echoing the findings presented by the current study. Female gender is a commonly identified risk factor that was not found to be significant in the current study.

The EAU catalogued risk modification strategies suggested by the various manuscripts included in their systematic review. Some strategies suggested include the appropriate use of antibiotic prophylaxis, broadened prophylaxis for pre-stented patients and longer operations, utilization of local resistance patterns when choosing antibiotics, and improved pre-operative counseling. In another review of infectious complications in stone disease, Wollin et al. highlighted the importance of identifying high-risk patients, treating active UTIs pre-procedure, and ensuring a negative PUC [14]. All of these recommendations are reinforced in the manuscript presented here.

Although there are many studies in the literature examining risk factors and predictors for infectious complications after URS for stone disease, most are single institution series and/or from academic medical centers which limits the generalizability of the results. The prospectively captured data from the current study include a diverse patient population and represent a variety of practice environments and practitioners, thus, making these findings more relatable and relevant. The work being done by MUSIC and ROCKS is truly important work and the authors are trailblazing in urology QI in the United States.
